# Analysis of Farmland Abandonment and Government Supervision Traps in China

**DOI:** 10.3390/ijerph18041815

**Published:** 2021-02-13

**Authors:** Yemei Li, Yanfei Shan, Ying Chen

**Affiliations:** 1 School of Economic Research, Shandong University, Jinan 250000, China; 161611173@csu.edu.cn; 2 School of Business, Central South University, Changsha 410083, China; yingchen0123@163.com

**Keywords:** farmland abandonment, government supervision trap, evolutionary game

## Abstract

Farmland abandonment has become relatively common in rural China. In the context of food security, the Chinese government has introduced policies for farmland abandonment supervision, but the effect of these policies has proven to be marginal. By constructing an evolutionary game model, our research explores the evolutionary logic during the supervision of farmland abandonment by governments and rural households. The results indicate that low food yield and high opportunity costs are the leading causes of farmland abandonment. The probable punishment administered by the central government for dereliction is a major motivation for the local government to practice farmland abandonment supervision. The low supervision avoidance cost for rural households leads local governments and households to form collaborations to jointly cope with central government supervision. When this occurs, local governments’ supervision of farmland abandonment falls into a trap, as it leads to continued supervision practices that are costly and ineffective. Food security risk comes from the contradictory population and land resources demands. To improve food security while managing these contradictory demands, it is both necessary and feasible for the government to control population growth and focus on farmland protection, whereas it is unnecessary and unfeasible for the government to supervise whether or not farmland should be abandoned.

## 1. Introduction

China is a country with a large population. As a result, farmland, agriculture and food safety issues have always been very important and have become one of the main concerns for the central government [1,2]. Since the COVID-19 (Corona Virus Disease 2019) outbreak began in late 2019, food supplies worldwide have been affected by the outbreak to a certain extent [3,4]. China, with sufficient food reserves and no food security problems, has been alert to food security risks in this context and pursued certain measures. With a large population base of 1.4 billion, the amount of farmland per capita in China is less than 40% of the global average [5]. However, in recent years, some of this farmland has been abandoned [6,7], which seems counterintuitive because additional demand still exists for agricultural product imports. Thus, there are several major questions that can be posed; why does farmland abandonment still occur? Furthermore, does the abandonment of farmland affect food security? The central government has imposed harsh penalties for “farmland abandonment”; does the government need to care about the abandonment of farmland? Are the policies that have been adopted appropriate for achieving farmland protection goals? The aforementioned questions require a scientific analysis of the reasons behind farmland abandonment, the nature of food security and how to ensure it is maintained as well as whether farmland abandonment affects food security, which would allow us to make a scientific and rational judgment.

**Figure 1:**
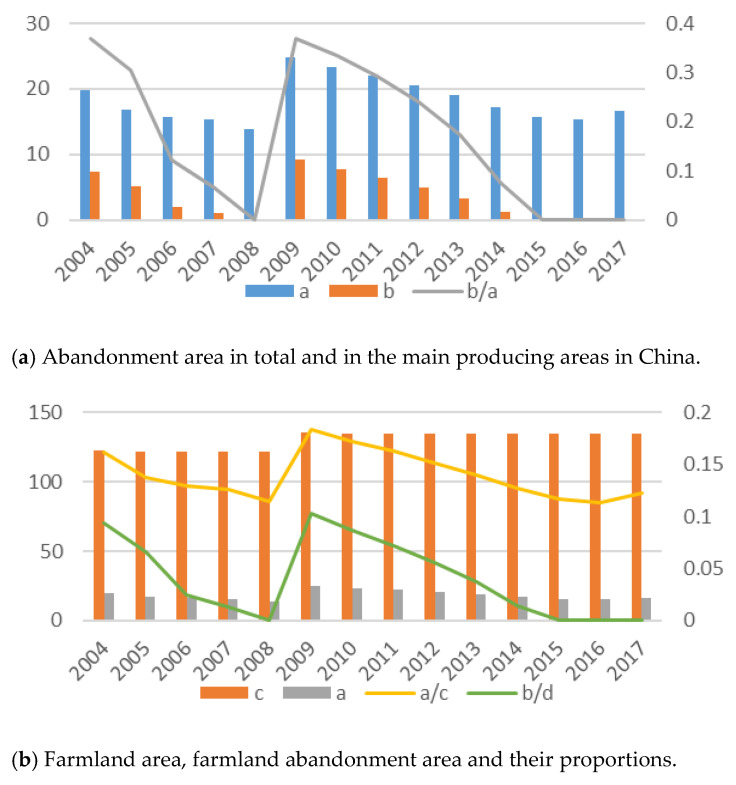
Farmland abandonment and its proportion of the total farmland area in China. (**a**) Note: a represents the total area of farmland abandonment; b represents the area of farmland abandonment in the main producing areas; (**b**) Note: c represents the total area of farmland; a represents the total area of farmland abandonment; b/d represents the farmland abandonment/farmland area in the main producing areas.

In China, farmland abandonment can be divided into two categories: farmland abandonment and half-farmland abandonment. Half-farmland abandonment refers to abandonment of prime farmlands which can be cultivated for two or three seasons but are only cultivated in one season. As to how many farmlands experience abandonment every year in China there are no accurate data. The first reason for this is that there is no index of farmland abandonment in the current statistical index system. The second reason is that it is difficult to define “the actual abandoned area” [8]. The third reason is that, with such a vast territory, comprehensive statistics are costly to obtain [9]. Using data from the National Bureau of Statistics of China, we obtained the farmland area and sown area of crops and calculated their difference to provide a rough estimate of farmland abandonment from 2004 to 2017 in China (as shown in Figure 1). In 2004, China established its land statistics system through a new Land Management Law. Since then, the farmland area in China has remained relatively stable. In particular after 2009, the Chinese farmland area has stabilized at about 1.35 million square kilometers. This indicates that the potential for increasing farmland is limited. From Figure 1, we can also see that from 2004 to 2017, the abandoned area of farmland fluctuated between 15 and 20 thousand square kilometers, accounting for about 15% of the total farmland area. This rough estimation confirmed the existence of farmland abandonment in China. Besides this, other researchers have provided factual confirmation of farmland abandonment [10,11,12].

There are two types of participants in farmland abandonment in China: first of all, rural households are the main participants in farmland abandonment. This kind of farmland abandonment is distributed all over the country. Although the farmland abandonment rate of each rural household is small, the total number across the entire country is considerable. Secondly, farmland abandonment by “large farmers” also takes place; such farmland abandonment is the result of unreasonable policies from the capital that affect the countryside, such as some capitalists collect lots of farmland from rural household to obtain state subsides but do not cultivate the land, causing farmland abandonment. Our research and the existing research mainly focus on the analysis of the first type of farmland abandonment. As part of existing research, Xu et al. [9] analyzed the relationship between labor migration and farmland abandonment in various provinces (and cities) of China and drew the conclusion that labor migration has a significant positive influence on farmland abandonment. Labor migration has also been analyzed in another research works [9,13]. With an economic model, Xie et al. explored the farmland abandonment mechanisms in Jiangxi Province; their results showed that the relevant variables associated with agricultural income are always correlated with farmland abandonment, such as regional industrialization, the opportunity cost of cultivating labor, the quality of farmland and so on [14]. Wang et al. also confirmed the importance of soil quality for farmland abandonment [15]. Shi et al. [16] explored the reasons for farmland abandonment in Chongqing and found the distance between townships to main urban zones, average net income per farmer and farmland area per farmer were significant factors. It is clear that the reasons affecting abandonment listed by Shi et al. also include the factor of farmers’ income. Besides, social capital and agricultural credit will also affect farmland abandonment through the opportunity cost of farming labor [16,17,18]. Above all, the income of rural households, including agricultural income and non-agricultural income, is the main direct factor affecting farmland abandonment. In this context, different researchers analyzed different factors of income, such as urbanization, labor migration, farmland quality, area and government policy [16,17,18,19,20,21,22,23,24,25,26]. Currently, under China’s land relevant management system, especially in the context of an inadequate rural social security system, rural households still have a “land complex”. With such a “land complex”, they would rather choose to abandon their farmland than to transfer the land away from themselves [27].

Based on the studies above, the causes of farmland abandonment are varied and include labor migration, industrialization, farmland quality, the distance between countryside and urban, average income, social capital, agricultural credit, and so on. However, the effects of these variables on households’ farmland abandonment choice, are ultimately transmitted through income. Urbanization shortening distance between city and countryside, increase the off-farm income, or the possibility of obtaining off-farm income. High income makes farmers tend to abandon less profitable farmland and engage preferentially in off-farm work. In addition, farmland quality, agricultural credit and other factors affect the level of farming income significantly as well. Hence, for analyzing the root causes of farmland abandonment, our study abstracts the factors influencing farmland abandonment into farming income and farming opportunity cost, through the phenomenon of labor migration. Our study innovatively introduces government supervision and its effectiveness into farmland abandonment research in China. For that, an evolutionary game analysis is used to explore the game choices of farmers and local governments. Chinese government’s legislation and mandatory regulation on farmland abandonment is a unique phenomenon. The basic background described as below.

In China, there is not a food security problem in the short term. Whereas, based on food security demand, basic state policy and the law of farmland protection, Chinese government’s supervision on farmland abandonment will be continued [28,29,30]. In 2004, in the second version of the “TRC (the People’s Republic of China) Land Administration Law”, reference was made to the provision for the collection of idle land costs in accordance with local standards. It is worthwhile to ask how effective the government’s policy measures are; from Figure 1, a slight decline in the amount of farmland abandonment during 2004 and 2005 can be observed, in particular involving permanent abandonment in non-food producing areas. After that, the policy effects have diminished. Farmland abandonment even increased in 2008. As a result, although the government has been promulgating policies to accelerate agricultural development and farmland protection in rural areas, the farmland abandonment problem has not been effectively solved so far [11,31,32]. However, the overall trend of farmland abandonment is in line with the broader economic trends in China [9,17,18]. Based on the above statistics and statement, it is clear that farmland abandonment is related to industrialization and urbanization. The government’s punishments are not only limited, but also short-term. Farmers’ circumvention of government punishments undermines the role of government policy. Finally, government supervision has fallen into the trap of ineffectiveness.

Based on the above background, our study uses an evolutionary game model to analyze the reasons for households’ farmland abandonment and the effectiveness of government supervision of farmland abandonment. Through analysis, we aim to make corresponding policy recommendations addressing farmland abandonment for China and other developing countries at similar stages of development. Specifically, the contributions of this paper can be summarized as follows. First of all, we use an evolutionary game model to analyze the mechanism of rural household farmland abandonment, which makes the study logic of the mechanism clearer and more scientific. Secondly, we analyze the effectiveness of government farmland abandonment supervision in our model; it is meaningful and innovative to study the influence of local government behaviors while under pressure from the central government on farmland abandonment. Last but not least, with numerical simulation, we simulate the evolution logic of the behavior choices of local governments and farmers under different extreme and realistic situations and draw corresponding conclusions to confirm the existence of the “government supervision trap” phenomenon described in the previous statistics.

The rest of the framework of this paper is organized as follows. Section 2 is the methodology, which contains the game payment function, replicated dynamic equations and evolutionary game equilibrium analysis. Section 3 is the numerical analysis, in which we analyze the equilibrium of households and the local government and the factors behind households’ or the local government’s strategic choices. In Section 4, the game results and discussion are described in detail. Conclusions and corresponding implications are provided in Section 5.

## 2. Model Building

### 2.1. Theoretical Framing Analysis

**Figure 2:**
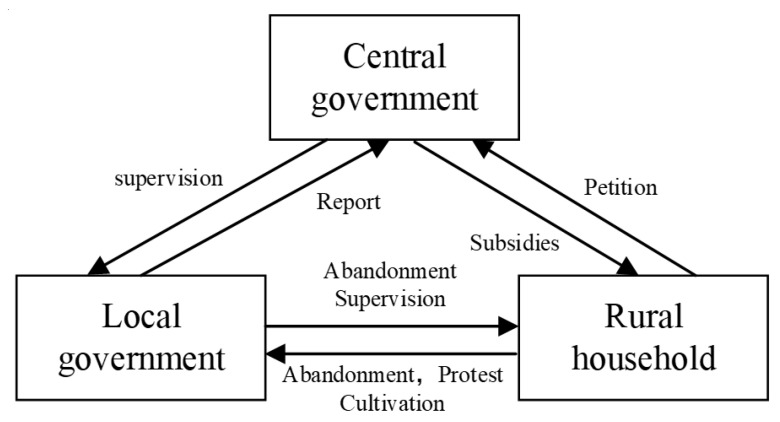
The game relationship between the central government, local governments and rural households.

Before we analyze the evolutionary game model, we will explain the relevant relationship in our paper, which is between the government and rural households during farmland abandonment incidents. In addition, it is necessary to discuss the role of the central government in the government supervision game, as shown in Figure 2. In the authoritarian system of China, the central government and local governments always form a trustee–beneficiary relationship in the process of policy implementation or information disclosure [33]. The central government is the principal actor; they are supposed to adopt effective incentives or relevant punishments to push local governments to be more diligent and act more responsively. In addition, under the structure of the authoritarian system of China, a top–down supervision system is often established to impel local governments to implement policies made by the central government; as our research indicates, this system includes the supervision of the behaviors of the local government’s supervision of farmland abandonment. Once local governments become involved in malpractice, the central government administers a relevant punishment. Commonly, the punishment for the local government and local government officials can be divided into two parts: fines and implicit punishments. Implicit punishments include personal reputation loss, damage to the offender’s political future, etc. [32].

Regarding the relationship between rural households and local government, the local government has two choices: to follow the instructions from the central government to conduct farmland abandonment supervision or to give up supervision but invest the associated funds in other performance-related projects. As for farmers, they have the right of farmland abandonment. It’s depends on their benefit-cost calculations. Actually, when the off-farm income is higher than the farmland income significantly, farmers are most likely to abandon farmland. Studies by Xu et al., Wang et al. and Shi et al. has confirmed the extensive farmland abandonment in rural China [9,15,16]. It is clear that when the local government chooses supervision, households may choose abandonment after considering the off-farm payoff.

On the other hand, the relationship between the central government and rural households includes subsidies and petitions, which affect local governments’ additional supervision costs. Since 1949, China has experienced the evolution from Control to Management to Pluralistic Governance. In governmental decision-making, public participation is gradually increasing. Citizens can participate in government decision-making through petitions, public opinion, social media, etc. ([32,33]). In our study, if farmland abandonment found by local government, and strictly punished, such as forfeiture, etc. Farmers may complain about the local government’s behavior through petitions. So that there is a certain probability of mass events’ outbreak, resulting in higher management costs (local government supervision thus becomes costly.) Those factors affect the choice of local government. What’s more, as for the central government, has stability and security as its top priority, may also rethink its administrative decisions. In addition, when households learn about the supervision, they may choose to sow crops but still undertake abandonment in essence, and then the supervision becomes ineffective. Above all, supervision is high-cost and inefficient. At the same time, once the local government abandon supervision, the punishment from the central government will be rather serious. In this regard, we need to clarify that, in our study, the main research objective is to investigate the causes of households’ abandonment and the effectiveness of local government supervision of farmland abandonment. Although our study involves three players, central government, local government and households. However, the complexity of the three-player game model may make the factor relationship between any two players less explicable. In addition, at this stage in China, the central government is unique in its decision making and urges local governments to take action by issuing instructions (“TRC Land Administration Law” in 2004; “Opinions on preventing “non grain” of cultivated land and stabilizing grain production” GBF [2020] No.44; “Guiding opinions on the overall utilization of abandoned farmland to Promote the Development of Agricultural Production” from the Ministry of Agriculture and Rural Affairs of the People’s Republic of China in 25 January 2021). The central government plays its main role mainly through administrative pressure on local governments. Given the purpose of this paper, and our study does not model the three-players in the model design.

### 2.2. Evolutionary Game Model

#### 2.2.1. Game Payment Function

Before the modeling, we need state that, our study focuses on the behavioral logic of farmland abandonment, and the effectiveness of local government regulation of farmland abandonment in China. So, the analytical framework of this question isn’t related with Chinese regional heterogeneity significantly. Therefore, to clarify and simplify our study, our modeling process does not provide a detailed dissection for that. Participants in the game: The two game parties in this model are the local government group and the household group, both of which are finite rational agents.

Participants’ behavior strategies: According to the evolutionary game theory, a member of the two groups is repeatedly drawn and paired into the strategy selection. The strategy set for the local government group is (supervise, not supervise), and the strategy set for the household group is (abandonment, no abandonment). The corresponding parameters are described in Table 1. Probabilities of behavioral strategy: At the initial stage of the game between the government and households, we assume that the probability of “household choosing to abandon farmland” is α(0≤α≤1), and the probability of selecting cultivation is 1−α. The probability of the government choosing supervision is β(0≤β≤1), and the probability of choosing no supervision is 1−β. 

In the game model, there are four sets of strategies for both the governments and the households: (1,1) represents the set of strategies in which the households choose to abandon farmland while the government chooses to supervise, (1,0) represents the set of strategies in which the households choose to abandon farmland while the government chooses not to supervise, (0,1) represents the set of strategies in which the households choose to cultivate farmland while the government chooses to supervise and (0,0) shows that the households choose to cultivate farmland while the government chooses not to supervise.

The total income from limited quantities of farmland makes it difficult for households to provide for the whole family, which is the main reason for households to abandon their farmland and engage in other non-farm jobs. With the growing attention on farmland abandonment in China, many provinces and autonomous regions have introduced administrative punishment supervisions to fine and pursue responsibility for farmland abandonment and ensure food security. In Zhejiang province, according to decree No. 106 of Zhejiang Provincial Government, penalties were expressly implemented as follow: “One who abandoned farmland under contracted management for two consecutive years, and when the original contracting unit did not terminate the land contract and withdraw land contracting rights, will be ordered to give the land contracting rights to the government, and will face a fine between 1000 yuan and 5000 yuan”. Since those local municipal policies have been implemented, it has become a crucial research question whether the implementation is reasonable or not, as well as its level of effectiveness and how the internal logic of policy implementation should be deduced. Based on that background, our study proposes the following basic hypothesis.

If a household abandons farmland, they will earn other income *r*_1_ from engaging in other non-farm industries, such as income from urban labor. At this point, the household will have to buy food in order to maintain their livelihood, assuming that the cost of the food is $p_{1}$. According to the relevant policy, the government should give registered households a certain amount of food subsidy S. When the government finds out that a household is still living in a rural area and has abandoned its farmland, according to local administrative penalties, the government has the right to impose a certain level of fines. The penalty here is the same value as the government’s gain from the penalty $r_{4}$. Given that households can avoid supervision at a lower cost, assuming the probability that households are found to have abandoned farmland is η, when a household chooses to cultivate farmland instead of abandoning it, they can earn a net income $p_{2}$ from food cultivation.

In addition, China has introduced a series of policies to reward food-producing households. The model is based on the following assumptions: (1) when the local government supervises farmland abandonment and no households choose to abandon farmland at the same time, then famers will be rewarded with a number of awards m; (2) when the local government supervises, the central government gives an incentive, which is set as *r*_2_; (3) if the local government chooses not to supervise farmland abandonment while households choose to abandon their farmland, the local government will be held accountable for their supervision by the central government. At this point, the expected cost is set as $c_{2}$, and as long as the local government chooses to implement supervision, there must be a behavior cost *c*_0_; when the government supervises and households abandon farmland, there is a government action to fine or confiscate farmland. If the government supervises and households abandon farmland, this can easily lead to mass incidents, resulting in additional costs, assuming that the expected value of this cost is $c_{1}$.

Based on the above assumptions, a matrix of benefits for households and the government under different behavioral choices involving farmland abandonment and supervision can be derived as shown in Table 2 below.

#### 2.2.2. Replication Dynamic Equations of the Government Supervision Game

From the return matrix mentioned above, the expected income of households who choose to abandon farmland and those who do not are $U_{1}$ and $U_{2}$, respectively, and the average income of the farming community is $\bar{U}$:

(1) $$U_{1}=\beta (r_{1}-p_{1}+s-\eta r_{4})+(1-\beta )(r_{1}-p_{1}+s)$$

(2) $$U_{2}=\beta (p_{2}+s+m)+(1-\beta )(p_{2}+s)$$

(3) $$\bar{U}=\alpha U_{1}+(1-\alpha )U_{2}$$

Likewise, the expected income of the government sector when it chooses to supervise and not to supervise are $V_{1}$ and $V_{2}$, (Equations (4) and (5))respectively, and the average income of the government sector group from the return matrix above is $\bar{V}$ (Equation (6)):

(4) $$V_{1}=\alpha (\eta r_{4}+r_{2}-c_{1}-c_{0})+(1-\alpha )(r_{2}-c_{0})$$

(5) $$V_{2}=\alpha (r_{3}-c_{2})+(1-\alpha )r_{3}$$

(6) $$\bar{V}=\beta V_{1}+(1-\beta )V_{2}$$

From evolutionary game theory, the replication dynamic equation for the households’ and government’s choice behavior is given by (Equations (7) and (8)):

(7) $$F(\alpha ,\beta )=\frac{d\alpha }{dt}=\alpha (U_{1}-\bar{U})=\alpha (1-\alpha )(U_{1}-U_{2})$$

(8) $$G(\alpha ,\beta )=\frac{d\beta }{dt}=\beta (V_{1}-\bar{V})=\beta (1-\beta )(V_{1}-V_{2})$$

#### 2.2.3. Evolutionary Game Equilibrium Analyses

Ordering dα/dt=0 and dβ/dt=0, solving the replication dynamic equation gives five local equilibrium points for this system, which are (0,0), (0,1), (1,0), (1,1) and $\left(\alpha^{*},\beta^{*}\right)$ (Equations (9) and (10)), where:

(9) $$\alpha^{*}=\frac{r_{2}-r_{3}-c_{0}}{c_{1}-c_{2}-r_{4}\eta }$$

(10) $$\beta *=\frac{r_{1}-p_{1}-p_{2}}{m+r_{4}\eta }$$

According to the method proposed by Friedman [17], the stability strategy (ESS) of the equation system can be derived from the local stability analysis of the Jacobi matrix (J) (Equations (11)–(14))of the system, where

(11) J=(∂F∂α∂F∂β∂G∂α∂G∂β)=(a11a12a21a22)

(12) $$\;a_{11}=\left(-1+2\alpha \right)\left(p_{1}+p_{2}-r_{1}+\beta \left(m+\eta \cdot r_{4}\right)\right)$$

(13) $$a_{12}=\left(-1+\alpha \right)\alpha \left(m+\eta \cdot r_{4}\right)$$

(14) $$a_{21}=\;\left(-1+\beta \right)\beta \left(c_{1}-c_{2}-\eta \cdot r_{4}\right)$$

(15) $$a_{22}=\;\left(-1+2\beta \right)\left(c_{0}-r_{2}+r_{3}+\alpha \left(c_{1}-c_{2}-r_{4}\cdot \eta \right)\right)$$

According to Friedman, when the determinant of the Jacobi matrix (Det J) of some equilibrium point satisfies Det J > 0 and (tr J) satisfies tr J < 0, then that equilibrium point is a locally asymptotically stable point in this system(Equation (16)); i.e., an ESS:

(16) $$\begin{array}{l} tr.J=\frac{\partial F(X)}{\partial X}+\frac{\partial F(Y)}{\partial Y}=a_{11}+a_{22}<0\\ Det.J=\frac{\partial F(X)}{\partial X}\frac{\partial F(Y)}{\partial Y}-\frac{\partial F(X)}{\partial Y}\frac{\partial F(Y)}{\partial X}\\ \;\;\;\;=a_{11}a_{22}-a_{21}a_{12}>0 \end{array}$$

Under different parameter constraints, the stability of the five local equilibrium points makes the game evolution process of both the government and households present different stable states, as shown in Table 3 below.

Obviously, $a_{11}+\beta_{22}=0$ is true at the equilibrium point, which does not satisfy the determinant conditions above. Thus, the equilibrium point $E_{5}=(\alpha^{*},\beta^{*})$ is obviously not an ESS. Therefore, four other evolutionary conditions need to be considered. The fourth subsection will analyze the stable point in those four conditions, as well as the influence mechanism of the corresponding factors.

## 3. Numerical Analysis

### 3.1. Equilibrium Analysis of Household and Government Behavioral Strategies

In order to clearly demonstrate the main evolutionary process of farmland abandonment and government supervision, MATLAB was used to simulate the dynamic evolution trajectories of different initial value points to their respective equilibrium points under different conditions. Drawing on the research results of Feng et al. [32], Friedman [33], Gao et al. [34,35] and Ginits [36] and taking the Chinese agricultural background into account, the variables of farmland abandonment and government supervision were valued to perform a numerical simulation. In conditions 1–4, the horizontal axis represents the time period (t) and the vertical axis represents the collaboration ratio (z) between the government (x) and the household; i.e., the firm (y) in our study.

Considering the vastness of China, there is a great variation among regions, the values are mainly based on the relative magnitude of each value in the major grain producing areas in central China. In Chinese central area, because of the small farmland per capita, the magnitude of the difference between farm return and off-farm returns, vary from year to year. In contrast, the Northeast area has a larger per capita farmland, higher total returns, and a relatively small amount of farmland abandonment, so the values in this subsection are not adapted to the Northeast food-producing region. Besides, for the western area in China, the poor quality of farmland, and low returns on cultivation. Because according to the differential land rent in economic theory, if most of the farmland is abandoned, it is not consistent with the evolutionary game process of farmers’ farmland abandonment and government supervision as well.

Based on the above analysis, combined with the facts of actual food producing areas in China, our study takes Changling Village, Zhajiang Town, Hengyang City and Hunan Province as a case for analysis.

The reasons for choosing this area are as follows. First, Hunan Province is located in the central area of China and is one of the main food-producing areas. The research base of Yuan Longping, a world-renowned rice expert, is located here. Therefore, this place has an important position in food security research in China and the world. Second, there is a demonstration area for Yuan Longping’s hybrid rice, which is of great importance to China’s food security strategy. On 2 November 2020, the average yield of the third-generation hybrid rice “Sanyou No. 1” developed by Yuan Longping’s team, reached a record high of 911.7 kg at the double-season late rice test site in Hengyang (reference from the official website of Hunan Provincial People’s Government in 3 November 2020, http://www.hunan.gov.cn/hnszf/hnyw/szdt/202011/t20201103_13949781.html, accessed date 11 February 2021). Thirdly, the hilly terrain of Changling Village in Zhajiang Town, Hengyang City, makes it difficult to implement large-scale mechanization. Its family farm has significant family co-production characteristics, which are consistent with the model characteristics of our study. Lastly, it serves as a fixed study area for our group, and data are easily available.

After the field survey and interviews with local farmers, we learned some actual situations. We can assume that a household has four members (parents and two children), then its income from food cultivation is roughly 5000 yuan. According to the calculation of dividing 1.2 mu (1 mu = 0.07 acres) of farmland per person, a family covers an area of 5 mu. Now, let’s calculate the average return of a mu farmland. The average rice harvest of a mu farmland is 850 kg, and the purchase price of rice is 2.4 yuan/kg. Subtract the costs as follows: harvester rental cost 150 yuan/mu, plow rental cost 140 yuan/mu, pesticide and herbicide cost 150 yuan/mu, fertilizer cost 150 yuan/mu, other field management cost 400 yuan/mu (including paddy field production environment inspection cost, drainage system construction and maintenance cost, human sprinkler irrigation cost, etc.), seed cost 50 yuan/mu. Then the average return of a mu farmland is roughly 1000 yuan. The value varies with household and other conditions. If the price of production materials rises, the total return will decrease; if the purchase price of grain rises, the total return will rise as well. If the household have only 1 member, the accounting will change again. (In addition, in different production teams, there are differences in the number of farmland per capita, such as some may be 0.8 mu/person). In this case, if the household chooses farmland abandonment, the cost of rice purchase is roughly 3000 yuan based on 5 yuan/kg. A household consumes 50 kg of rice in a month and about 600 kg of rice in a year. In addition, according to China’s food cultivation subsidy policy, the farmland subsidy is 100 yuan per mu. Therefore, the subsidy for a 5-mu field is 500 yuan. As for the non-farm income, the non-farm income of different households varies greatly due to the different ability of household members. If all the members of a household are adult workers who work outside, such as engaged in urban construction, the annual net income of four people can reach more than 10,000 yuan. If there are two children in the household to be supported, and one adult worker (elders, women etc.) stays to take care of the children, then the annual income can hardly reach 8000 yuan. The values of the number of government fines are taken from the executive orders published by Zhejiang Province (decree No. 106 of Zhejiang Provincial Government). According to the TRC Land Administration Law issued in 2004, the fine for farmland abandonment is 1000–5000 yuan. It has provided local governments with the power to impose fines for abandonment of cultivated land, it does not provide details on the specific discretion of local governments. Hunan provincial government has not issued a document about specific fine amount in public. For the convenience, the numerical simulation part of our study mainly refers to the penalty regulations of Zhejiang Province and other provinces.

The numerical values of the cost of government supervision and its opportunity cost are taken from the estimation of the cost of supervision in the information disclosure documents of each local government. In addition, given the diversity of other indicators in practice, they will not be elaborated here. To simplify the analysis and express the evolutionary process clearly, the relevant data units in the following of our study are all in thousands of yuan, and the data units are no longer emphasized. Based on the above data background, this subsection will first analyze the four equilibrium points of our study’s model.

**Figure 3:**
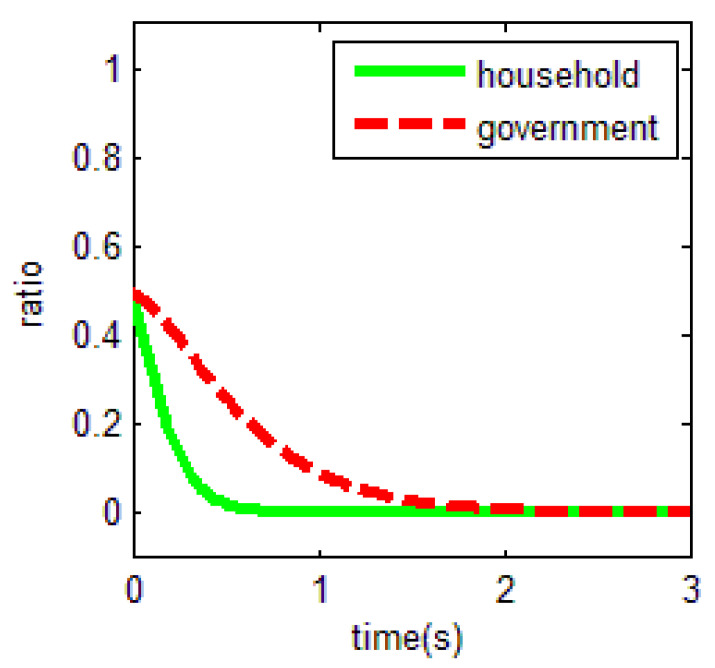
Evolutionary equalization of (0,0).

From Table 3 and the Jacobi determinant condition, the ESS chosen by the household and the government is $E_{1}=(0,0)$, when $-p_{1}-p_{2}+r_{1}<0$ and if $-c_{0}+r_{2}-r_{3}<0$. In this case, households choose not to abandon farmland and the government chooses not to supervise. We assume that parameters $p_{1}=3,\;p_{2}=5,\;r_{1}=1,\;c_{1}=1,\;m=0.5,\;\eta =0.5,\;r_{2}=2,\;r_{3}=4,\;c_{2}=3,\;r_{4}=1,$ $c_{0}=0.5$, meet the above conditions. To verify the validity of the model, the initial evolution ratio of the government and household is set to 0.5. Thus, the evolutionary process of households and government in this condition is obtained as shown in Figure 3.

Based on the above values, it is easy to find that the key for households not to abandon farmland is $r_{1}=1$. That is, when farm households have difficulty in obtaining off-farm income or have few opportunities to work outside the farm, they tend to choose to cultivate the land. In an existing study, Shi et al. [16] found the distance between townships to main urban zones were significant factors of farmland abandonment. Our study also confirms the evolutionary results of this paper, that is, the size of the opportunity for off-farm benefits is crucial for farmers to choose whether to abandon farmland. In the case of the government, the uneconomical behavior will make it quit farmland abandonment supervision.

As a result, the equilibrium solution holds when the households’ income from engaging in nonfarm industries is higher than the difference between the net income from food cultivation and the cost of buying food. This is because as rational people, the households will choose farmland abandonment in order to obtain a higher income; for the government, if the government chooses not to supervise farmland abandonment, it can use this funding in other ways, such as for investment incentives. When the maximum benefit generated by this behavior is greater than the difference between the maximum benefit of supervision and the expected cost of supervision, the government will be inclined to give up supervision. Thus, those are important factors in behavioral decisions both regarding the off-farm benefits generated by households’ farmland abandonment and the opportunity costs of the government’s choice of supervision.

**Figure 4:**
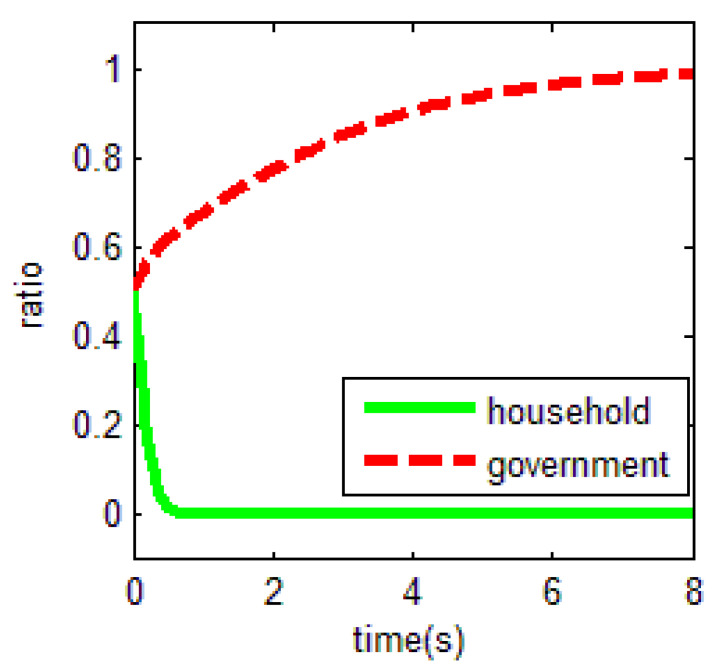
Evolutionary equalization of (0,1).

From Table 3 and the Jacobi determinant conditions, the stability strategy (ESS) chosen by both the household and the government is $E_{1}=(0,1)$, when $-m-p_{1}-p_{2}+r_{1}-\eta r_{4}<0$ and $c_{0}-r_{2}+r_{3}<0$. At this point, the household chooses not to abandon farmland and the government chooses to supervise farmland abandonment. We assume that the parameters $p_{1}=3,\;p_{2}=5,\;r_{1}=1,\;c_{1}=1,\;m=0.5,\;\eta =0.5,\;r_{2}=2,\;r_{3}=1,\;c_{2}=3,\;r_{4}=1,\;c_{0}=0.5$ meet the above conditions. To verify the validity of the model, the initial evolution of both the government and the household is set to 0.5, and the evolution of the household and the government in this case is shown in Figure 4. Based on the above values, $c_{2}=3$ is a key factor for the government to choose the supervision of farmland abandonment. This confirms the central government policy in China nowadays. The central government attaches great importance to farmland abandonment supervision for imposing high penalties relatively. This situation may affect the cost-benefit calculation rule of local governments to some extent.

As a result, the equilibrium solution holds when the difference between the net income of households in non-farm industries and the net income of government supervision is smaller than the net income in food cultivation, when government supervision is in place. In other words, in the case that households do not abandon farmland, the overall income obtained by not abandoning farmland is greater than that of farmland abandonment. The government will choose to supervise when the cost of forfeiture is much smaller than the difference between the benefits and opportunity costs of government supervision.

This equilibrium is the optimal equilibrium that the society expects to achieve. However, it is quite unachievable. In particular, for the government, the choice of the government’s supervision strategy depends on lower supervision costs *c*_1_.

From Table 3 and the Jacobi determinant conditions, the stability strategy (ESS) chosen by both the household and the government is $E_{1}=(1,0)$ when $p_{1}+p_{2}-r_{1}<0$ and $-c_{0}-c_{1}+c_{2}+r_{2}-r_{3}+r_{1}+\eta r_{4}<0$. In this case, the household chooses to abandon farmland and the government chooses not to supervise. We assume that the parameters $p_{1}=3,\;p_{2}=5,\;r_{1}=9,\;c_{1}=9,\;m=3,\;\eta =0.5,\;r_{2}=2,\;r_{3}=4,\;c_{2}=10,\;r_{4}=5,\;c_{0}=3$ meet the above conditions.

**Figure 5:**
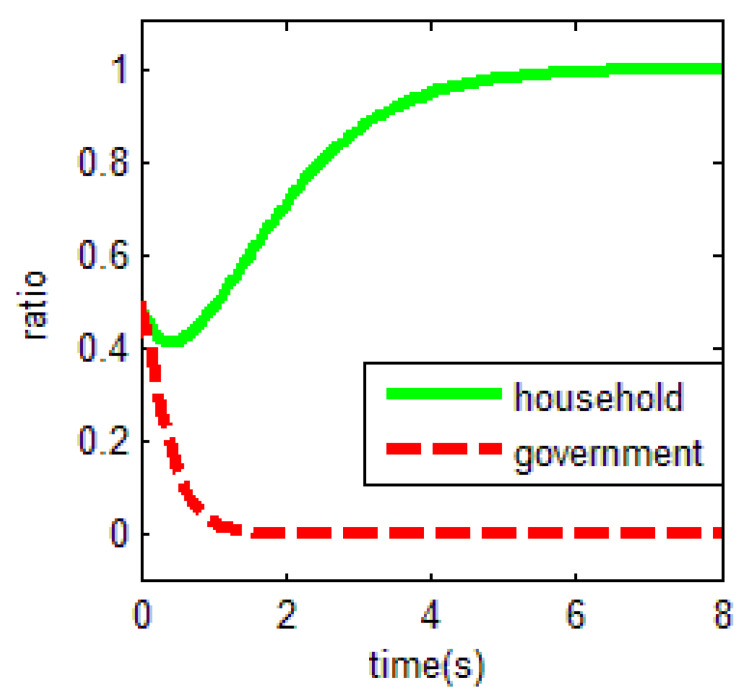
Evolutionary equalization of (1,0).

To verify the validity of the model, the initial evolution of both the government and household is set to 0.5. The evolution of the household and government in this case is shown in Figure 5 below. To some extent, the above values represent the influence of mass incidents on local government decision-making. When mass incidents occur frequently in a region and people’s consciousness of safeguarding rights is high, the decision of local government is affected by its possibly extremely high execution cost.

Thus, the equilibrium solution holds; when the households’ net income from food cultivation is less than the difference between non-farm industry income and the cost of buying food, by a cost–benefit estimation, the households will choose to abandon farmland. As for the government, the benefits of government supervision are smaller than the costs of government supervision; i.e., on the one hand, the sum of incentives and fines gained from farmland supervision is less than that of not performing supervision, while on the other hand, the expected loss for not supervising farmland is smaller than the sum of opportunity costs and forfeiture costs when the government performs supervision. In this case, the government tends to give up supervision.

From the above hypothetical conditions, households’ low income from food cultivation is the root cause of most farmland abandonment. The limited farmland and low total income make it difficult to obtain a high income from cultivation. As for the government, the low cost of evading supervision, such as migration to the city, makes the cost of supervision high, while the benefits from forfeiture are nearly 0. Thus, although the local government chooses to comply with the will of the superior government to supervise under certain conditions, this political compliance is difficult to maintain under the conditions of differences in revenue costs. As a result, the strictness of their supervision behavior usually follows the characteristic political cycle.

**Figure 6:**
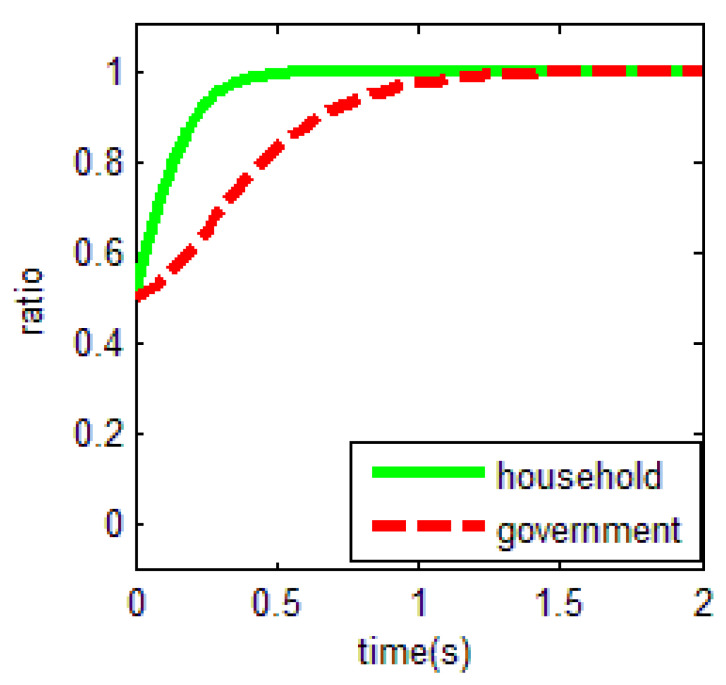
Evolutionary equalization of (1,1).

From Table 3 and the Jacobi determinant conditions, the stability strategy (ESS) chosen by both the household and the government is $E_{1}=(1,1)$ when $m+p_{1}+p_{2}-r_{1}+\eta r_{4}<0$ and $c_{0}+c_{1}-c_{2}-r_{2}+r_{3}+r_{1}-\eta r_{4}<0$. In this case, the household chooses to abandon farmland and the government chooses to supervise farmland. We assume that the parameters $p_{1}=3,\;p_{2}=5,\;r_{1}=20,\;c_{1}=5,\;m=0.5,\;\eta =0.5,\;r_{2}=2,\;r_{3}=4,\;c_{2}=9,\;r_{4}=5,\;c_{0}=0.5$ meet the above conditions. To verify the validity of the model, the initial evolution of both the government and the household is set to 0.5. The evolution of both the household and government in this case is obtained, as shown in Figure 6 below.

This is the most widespread scenario in Chinese central area in recent years. Based on the cost-benefit ratio, households abandon their farmland and work outside. Then the local governments choose to supervise the inefficient farmland abandonment under pressure from the central government. They have gradually formed a conspiracy to respond to the central government’s policies.

The equilibrium solution holds if, in the (0,1) case and the (1,1) case, when households’ total income from farmland abandonment is much greater than the sum of households and the government’s total benefit from cultivating farmland, the household tends to abandon farmland. For the government, compared with the (1,0) case, when the benefits of government supervision are much greater than the costs of government supervision, the government tends to supervise farmland. Similar to for the (0,1) case, the key to the government’s choice of supervised behavior is the lower supervised costs. In the real situation, due to the higher opportunity cost and lower supervised benefit of government supervision, the lower c_1_ is difficult to achieve, then the government tends to choose to abandon supervision.

### 3.2. Analysis of Factors Influencing Households’ Behavioral Strategies

In this subsection, the main influencing factors of households’ behavioral choices are analyzed. According to the equilibrium conditions shown in Section 3.1, the off-farm income of households, the net income from households’ food cultivation and the additional incentives for food cultivation received by households from incentive policies are the influencing factors behind households’ behavioral strategic choices. The government has no right to impose fines on households who have substantially abandoned but not formally abandoned farmland; for example, households can sow seeds randomly and households who work outside can move to cities, and therefore household penalties *f* for farmland abandonment do not work. Therefore, a sensitivity analysis is not conducted for that instance.

Firstly, in view of the effect of nonfarm returns on farmland abandonment, in the target areas examined, there are large differences across households, and across years for the same household. Households with high labor force share can obtain higher nonfarm returns. During the upward phase of economy, more nonfarm jobs are created. There is a large gap between supply and demand for urban jobs, which provides opportunities for farm households. The labor force in the target study area, Zhajiang Town, Hengyang City, mainly flows to Guangdong Province for work. Since the COVID-19 in 2020, a number of small-size enterprises bankrupt, and wages are much less than that in 2019. Therefore, some households return to the countryside. There is a slight decrease in farmland abandonment.

**Figure 7:**
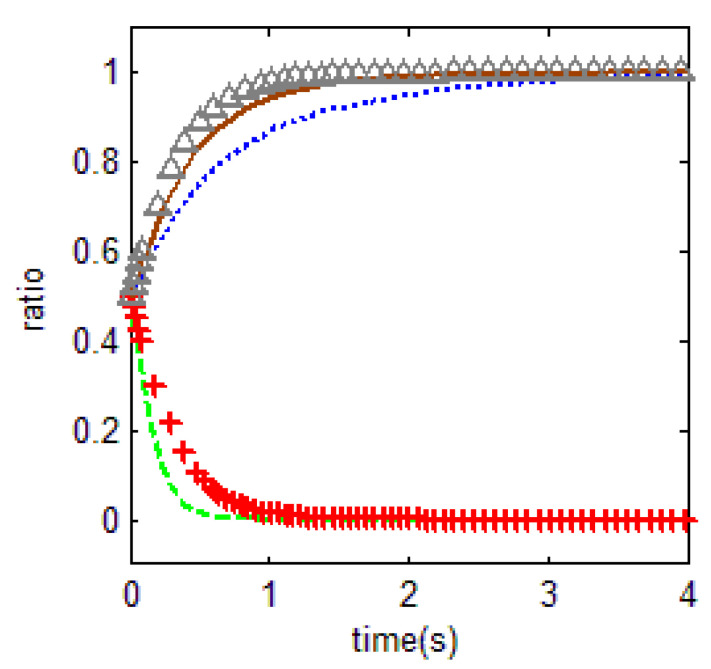
Influence of off-farm earnings on behavioral selection.

Following relevant research [34] and field survey results in Changling village, in Figure 7, the values of the relevant parameters are $p_{1}=3,\;p_{2}=5,\;c_{1}=5,\;m=0.5,\;\eta =0.5,\;r_{2}=2,\;r_{3}=4,\;c_{2}=9,\;r_{4}=5,\;c_{0}=0.5$, where $r_{1}=1,\;5,\;12,\;13,\;14$ (in Figure 7, from the bottom to the top, the green line shows $r_{1}=1,$ the red line shows $r_{1}=12,$ the blue line shows the brown line shows $r_{1}=13,$ and the gray line shows $r_{1}=14$), to explore the impact of changes in households’ non-farm income on their behavioral choices. From the figure above, it is easy to conclude that farm households increasingly tend to abandon farmland along with the increase of their non-farm income. The evolutionary equilibrium of households’ behavior is towards cultivation when the households’ nonfarm income is 1 and 5. The evolutionary equilibrium of households’ behavior is abandonment when households’ non-farm income is 12, 13 and 14, and the evolutionary time to equilibrium is gradually shortened.

**Figure 8:**
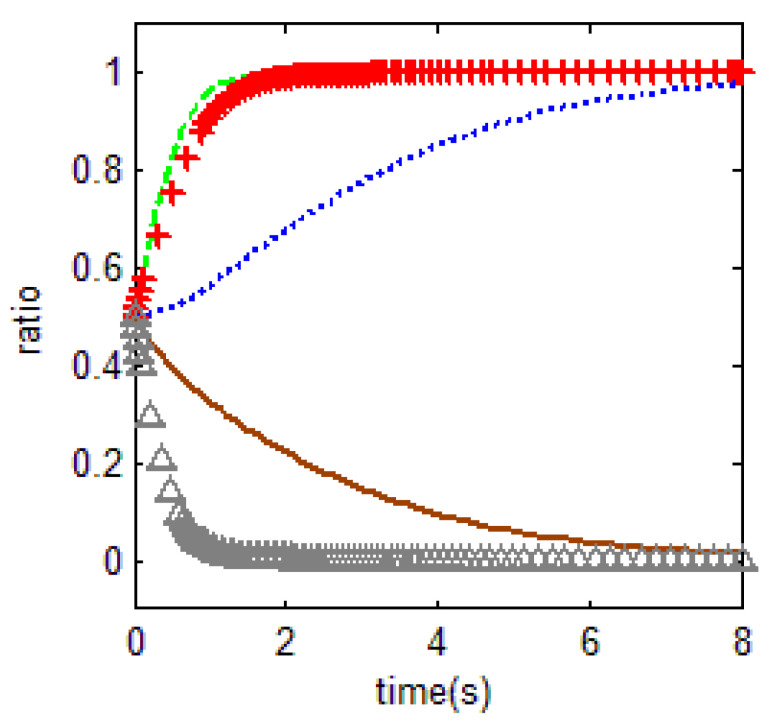
Influence of farmland yield on behavioral selection.

Figure 8 shows the influence of the farming return on the households’ choices. According to the field survey, there are many factors that affect the returns in family farming. Specifically, the amount of households’ farmland, the purchase price of food, and the price of production materials, and so on.

In the case of Changling Village, a household’s farmland amount varies greatly from different produce group in China (a kind of local form of production organization). Some groups are 1.2 mu/person, while others are 0.8 mu/person. Under the effect of incremental returns to scale, the more farmland, the higher the farming income. As for the purchase price of grain in Changling village of Hengyang city was 4.3 yuan/kg in 2018, while the highest purchase price of grain reached 5 yuan/kg in 2020.

The values of the relevant parameters in Figure 8 above are $p_{1}=3,\;r_{1}=7.5,\;c_{1}=4,\;m=0.5,\;\eta =0.5,\;r_{2}=2,\;r_{3}=4,\;c_{2}=3,\;r_{4}=1,\;c_{0}=0.5$ The household’s food yield takes the values of $p_{2}=1,\;2,\;4,\;5,\;8$ and it is clear that the evolutionary direction of Figure 8 is mirror-symmetric to Figure 7, which is exactly the opposite. When $p_{2}=1,\;2,\;4$ the household’s evolutionary equilibrium strategy is to abandon farmland. However, with the increase of household’s food income, when $p_{2}=5,\;8$ the household’s equilibrium choice is food cultivation to obtain farm income. It is clear that, even if the household’s evolutionary equilibrium strategy has not changed, with the increase in the household’s food income, the situation of farmland abandonment is reduced while the tendency of food cultivation is increased. Obviously, even if the households’ evolutionary equilibrium strategy remains unchanged, with the increase of their food cultivation income, they are less likely to abandon farmland. As a result, households tend to keep their farmland instead of abandoning it when their net farm income increases. Households’ net farm income, as the opportunity cost of abandoning farmland, is obviously substitutionally related to the change of households’ off-farm income. There is also a substitution effect on behavioral choice.

**Figure 9:**
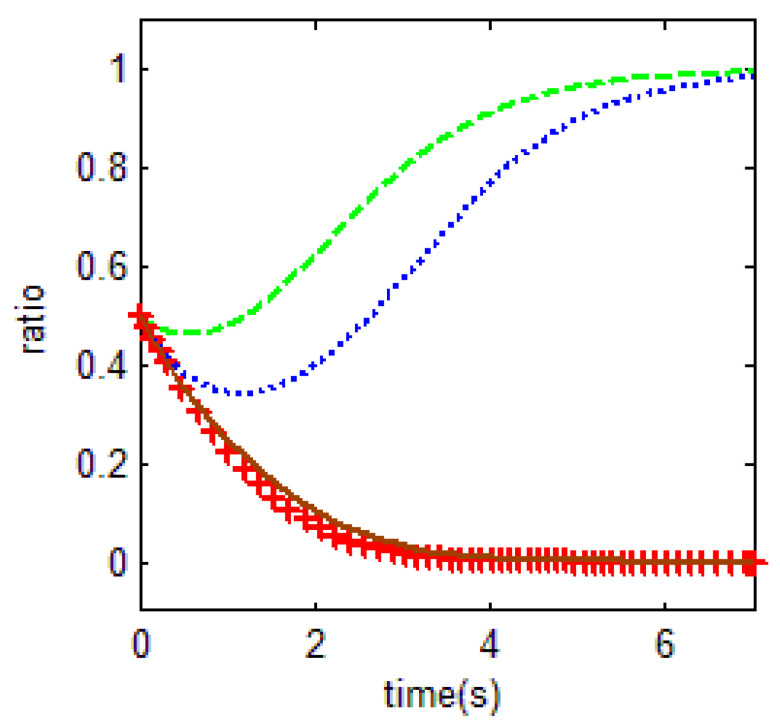
Influence of food incentive on behavioral selection.

Next, the effect of government incentives on households’ behavioral choices under a food cultivation incentive policy is explored. In practice, the incentives for farming are generally low, but may be increased in some specific years. In 2020, the Hengyang city followed the central government’s instruction to provide incentives for double-season rice cultivation. Nonetheless, the contribution of grain cultivation incentives remains low to total income. The values of the relevant parameters in Figure 9 above are $p_{1}=3,\;p_{2}=5,\;r_{1}=9,\;c_{1}=7,\;\eta =0.5,\;r_{2}=1,\;r_{3}=1,\;c_{2}=3,\;r_{4}=5,\;c_{0}=0.5$. Considering the magnitude of the correlation between households’ income from food cultivation incentives, the government’s incentive coefficient is set as m=0.5,2.

The influence of cereal growing rewards on behavior choices is shown in Figure 9. From top to bottom, when m=0.5, the red line shows the evolutionary path of the government and the green line shows households’ behavior; when m=2, the brown line shows the evolutionary path of the government and the blue line shows households’ behavior. Regardless of whether *m* takes the value of 0.5 or 2, over time, households eventually choose to abandon farmland. Keeping the other variables unchanged, the government and households are caught in a stable dynamic game as *m* increases.

Regarding the evolution of the probability of governments’ supervision from 0.5 to 0, the probability of households’ abandonment gradually increased to 1. As we found out from the above values, despite the government incentives for farming is increased. The increase in incentives for farmers to grow food has a negligible impact on their total income increase compared to their higher off-farm returns (i.e., opportunity cost of growing food). Thus, in Figure 9, as m increases from 0.5 to 2. The evolution of households’ farmland abandonment slows down over time. Meanwhile, the evolution of government supervision abandonment slows down as well. Ultimately, the increase in the incentive for farming did not change the evolutionary outcome fundamentally. Based on the above analysis, the factors influencing the evolution of households’ behavior are summarized in

**Conclusion** **1:** *Increasing households’ income from food cultivation is the most effective way to reduce the probability of farmland abandonment behavior, and the opportunity cost of cultivating food is the main reason why households abandon farmland. The additional incentives to households discussed previously cannot reverse the behavioral choices of households*.

### 3.3. Analysis of Factors Influencing Government Action Strategies

On the basis of the equilibrium conditions analysis in Section 3.1, the main factors influencing the choice of government behavior are analyzed in this subsection. According to the equilibrium conditions in Section 3.1, to take the value of the characteristics, the government supervision costs $c_{1}$, government supervision opportunity costs $r_{3}$ and the expected loss $c_{2}$ from the government not performing supervision are selected as the influential factors in the choice of the government’s strategic behavior.

As shown in Section 3.1 and Section 3.2 above, due to the low cost for households to avoid supervision and the many ways to cope with supervision, high actual costs of government supervision $c_{1}$, such as the technical input of mobile remote sensing technology and mass events such as violent resistance, may occur in the process of supervision and enforcement. In the case of a relative high value of $r_{3}$, a higher income may be obtained by investing the supervision costs in other industries to promote local economic development. Thus, local government supervision is not economically rational. The local government chooses to supervise because of the performance appraisal from the central government and the penalties $c_{2}$ for deficient supervision imposed by the latter party. This subsection will analyze the impact of the three influences above on the evolutionary behavior of the government.

**Figure 10:**
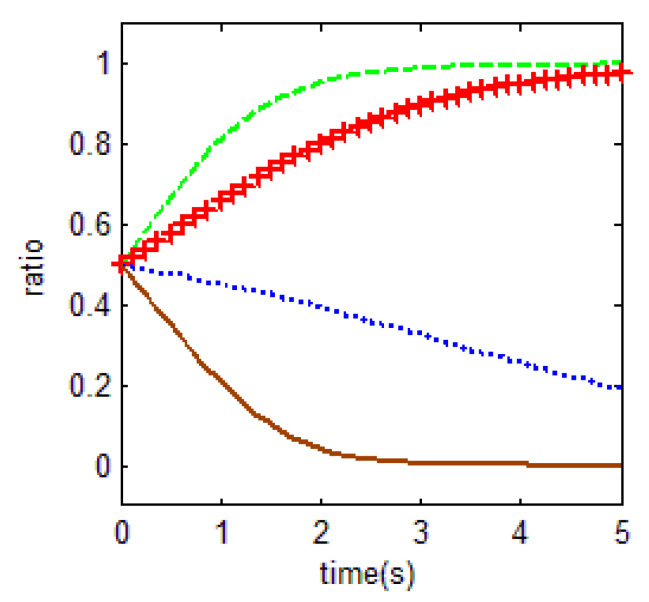
Influence of supervision cost on behavior selection.

**Figure 11:**
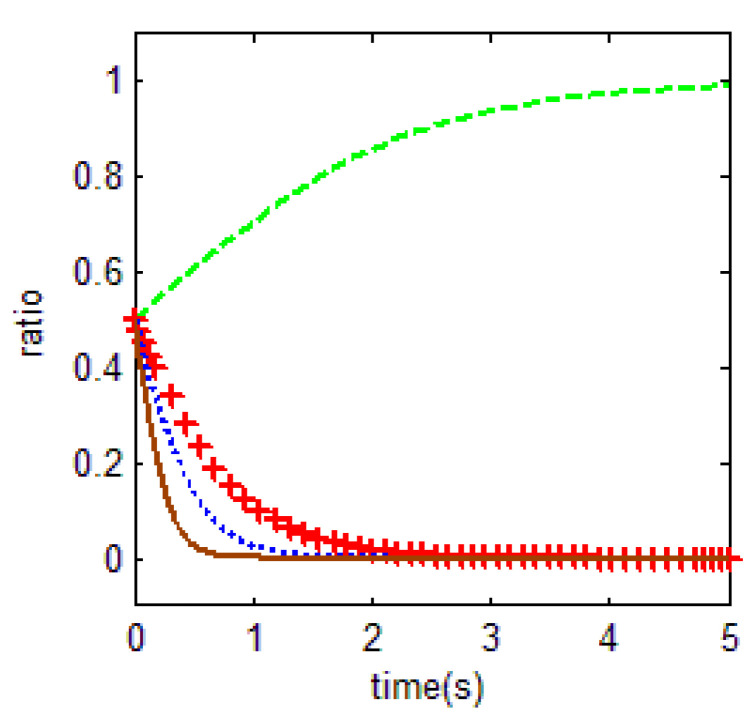
Influence of opportunity cost on behavior selection.

The cost of supervision conflict is relatively high in these research sites. The general public’s high enthusiasm in petitioning and complaining, greatly increases the relative magnitude of the problem. In Figure 10 below, the values of the relevant parameters are $p_{1}=3,\;p_{2}=5,\;r_{1}=9,\;m=0.5,\;\eta =0.5,\;r_{2}=1,\;r_{3}=0.8,\;c_{2}=2,\;r_{4}=1,\;c_{0}=0.1$, where $c_{1}=0,\;1.5,\;3,\;5$ (In Figure 10, from top to bottom, the green line, the red line, the blue line and the brown line shows $c_{1}=0,\;1.5,\;3,\;5$, respectively). It is clear that, with the increasing supervision cost, the probability of changing government supervision decreases. The evolutionary equilibrium of government behavior regarding supervision gradually evolves to the abandonment of supervision. The evolutionary trend of Figure 11 is similar to that of Figure 10.

For the local government in Hengyang, if does not supervise farmland abandonment, then it can apply the funds to attract investment. By introducing preferential policies and improving the business environment, it is obviously easier to achieve political success. In Figure 11, the values of the relevant parameters are $p_{1}=3,\;p_{2}=5,\;r_{1}=9,\;c_{1}=1.8,\;m=0.6,\;\eta =0.5,\;r_{2}=1,\;c_{2}=2,\;r_{4}=1,\;c_{0}=0.5$,where $r_{3}=0,\;3,\;4.5,\;8$ (in Figure 11, the green line, the red line, the blue line and the brown line show $r_{3}=0,\;3,\;4.5,\;8$ respectively). In Figure 11, when $r_{3}=0,\;3,\;4.5$, the government’s evolutionary equilibrium strategy is to perform supervision. As the opportunity cost of supervision increases, the government gradually abandons supervision.

**Figure 12:**
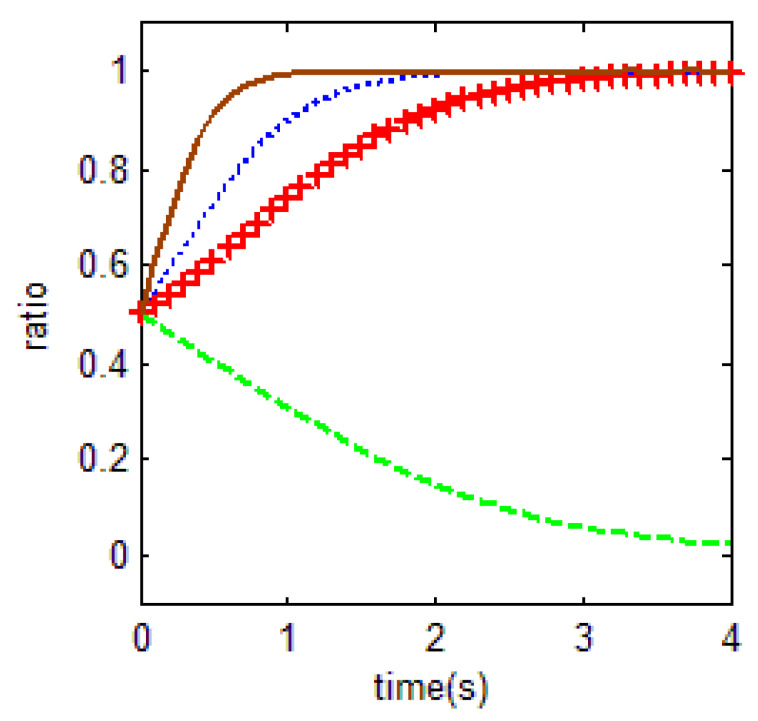
Influence of dereliction of duty cost on behavior selection.

Given that Hengyang has been a demonstration base for Yuan Longping’s hybrid rice, the central government is extremely concerned about the implementation of food security strategies in this area. The local government has also responded to the central government’s instructions. The fields are covered with slogans to prevent the abandonment of farmland. In Figure 12 above, the values of the relevant parameters are $p_{1}=0.5,\;p_{2}=1,\;r_{1}=3,\;c_{1}=1,\;m=0.5,\;\eta =0.5,\;r_{2}=1,\;r_{3}=1,\;r_{4}=1,\;c_{0}=0.5$, where $c_{2}=0,\;3,\;5,\;10$ (in Figure 12, the green line, the red line, the blue line and the brown line shows the values $c_{2}=0,\;3,\;5,\;10$ respectively). Obviously, the increase in the cost of the local government’s dereliction of duty significantly affects the local government’s behavioral choice. As the cost of its dereliction of duty increases, the local government’s strategy choice gradually evolves from abandoning supervision to supervision, and the evolution is fast and upward. This leads to conclusion 2 regarding the influence of the government’s behavior strategy.

**Conclusion** **2:** *Both the cost of supervision and opportunity costs are important factors influencing the choice of the government’s supervision behavior. Due to the high cost of supervision, the local government mostly abandons supervision, while the high penalties imposed by superior government for the local government’s dereliction of duty are the root cause of deviating from social welfare maximization*.

### 3.4. Analysis of Special Cases in a Realistic Situation

#### 3.4.1. Analysis of Government Supervision Traps

Section 3.1,Section 3.2,Section 3.3 presented an analysis of the behavioral choices and influencing factors of government and households at different stages and contexts. Below, we explore the specific stage of the game process between households and the central government in China where the level of abandonment is serious, and the evolution of the game behavior of households and the government occurs.

**Figure 13:**
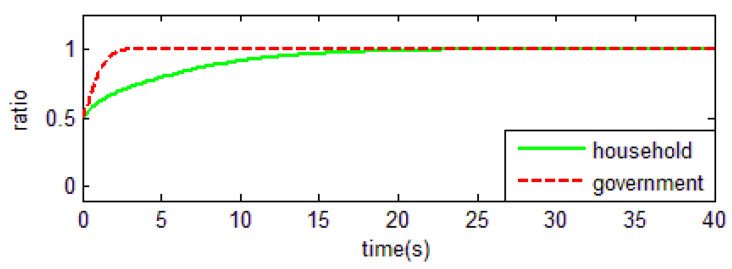
Government supervision traps in a realistic situation.

In Figure 13 below, the green line shows the evolutionary process of households and the red line shows the evolutionary process of the government.

Currently, the main reason for serious levels of farmland abandonment in areas of China is that the farmland area of a single household is too small; this means that the total income from farmland is insufficient to provide for an entire family, while households engaged in other non-farm industries obtain a higher income, meaning that the opportunity cost of food cultivation is high, thus setting the two key variables as $r_{1}=9$. For the government, combined with the previous analysis, the cost of supervision is relatively high, firstly because of the high cost of basic equipment, secondly because of the high risk of mass incidents, which affects the stability of the region, and thirdly because it is easier for local households not only to cope with supervision but also to move their families to towns and cities to avoid fines.

In addition, the opportunity cost of government supervision $r_{3}$ is set to 4. The loss is set at 10 if the local government fails to supervise the farmland abandonment and is discovered by the central government. The above two settings are consistent with the practice that local governments are ineffective in farmland abandonment supervision, and the central government force local governments to do so by promotion, talking, and warning officials in order to implement food security strategies. Besides, the other variables are specified as $p_{1}=3,\;r_{2}=1,\;r_{3}=2,\;c_{2}=10,\;m=0.5,\;\eta =0.3,\;r_{4}=1,\;c_{0}=0.5$. As for the government, the high cost of supervision is partly due to the large investments needed in equipment and technology, because it is easy for supervision to trigger mass incidents and affect regional stability and finally because the cost of households avoiding supervision is low. Thus, we obtain Figure 13, which shows that the probability of being punished by superior government increases when the local government’s dereliction of duty leads to serious farmland abandonment; thus, the government has to supervise farmland. As government supervision increases, households will reduce their level of farmland abandonment, such as by performing the symbolic sowing of seeds. As a result, the government continues to enforce supervisions, while households essentially sow seeds to cope with the supervisions. Thus, the government and households gradually form a collaboration, affecting the overall welfare of society. This paper emphasizes that the high cost of government supervision, especially the difficulty in identifying the abandoning behavior, is an important reason for farmland abandonment. Based on this, caused by technological progress, the impact of increasing farmland abandonment detection probability on government and farmers’ behavior, will be analyzed in our study. The analysis of variable η is as follows. In order to study the evolution of government and household behavior, when the government’s supervised capacity changes, the probabilities of households’ abandonment being discovered and the households receiving fines are set to 0.01 and 0.8, respectively.

**Figure 14:**
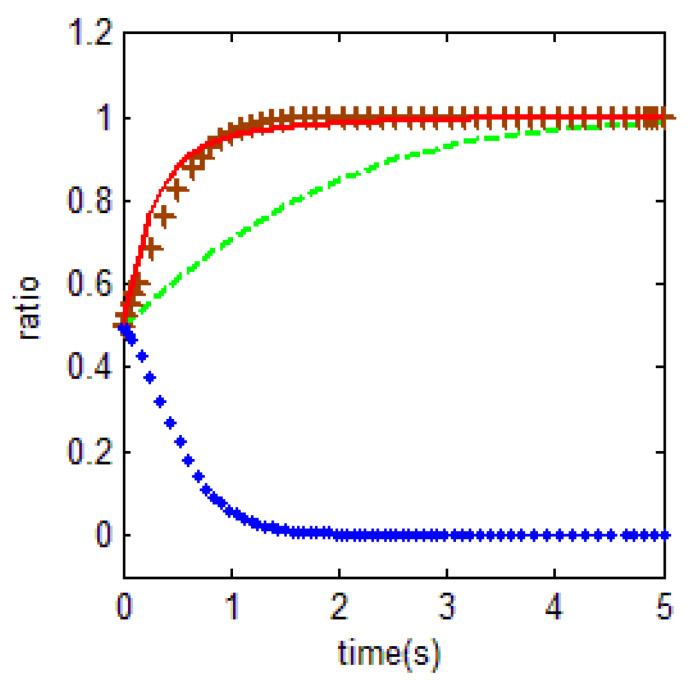
Influence of supervision technology on behavior selection.

In Figure 14, the green dashed line is the choice of the farmer at η=0.01, and the brown line is the choice of the local government at this time. The blue line is the choice of the farmer at η=0.8, and the red line is the choice of the local government in this time. Other values taken are $p_{1}=3,\;p_{2}=5,\;r_{1}=9,\;c_{1}=1.5,\;m=0.1,\;r_{2}=4,\;r_{3}=2,\;c_{2}=5,\;r_{4}=1,\;c_{0}=1$. As can be observed from Figure 14, when the probability of households’ abandonment being discovered increases from 0.01 to 0.8, the behavioral choice of households evolves from abandonment to non-abandonment. Due to the supervision of higher-level government, the local government behavior changes to supervision in both cases. It is clear that the evolution is faster when the probability of penalty is increased to 0.8. Obviously, the probability of punishment can promote government supervision. In reality, the low cost of households avoiding supervision in China make the probability of being found to have abandoned farmland extremely low. In addition, farmland serves as a survival guarantee for households, meaning that government fines are difficult to enforce. With the improvement of technology, *η* has the tendency to improve. To a certain extent, this will improve the efficiency of farmland utilization. 

Based on the above, Conclusion 3 is obtained:

**Conclusion** **3:** *Due to the high supervisory costs of the government as well as the low costs of evading supervision, and the fact that farming income is currently difficult to improve, increases in the severity of the local government forfeiture under government supervision will not solve the current situation of farmland abandonment. On the contrary, with the evolution of the game, the local government and abandoned households will tend to form a collaboration to deal with superior government supervision. Government supervision thus falls into a trap, eventually reducing the overall level of social welfare*.

#### 3.4.2. Analysis of the Two Behaviors under Great Changes in External Circumstances

The model can analyze the dramatic external environment changes as well. For example, in 2020, when the novel coronavirus outbreak, some countries closed their food export channels. As a result, under the background of not major changes in food demand, China was forced to reduce food supply, Chinese food market showed supply less than demand at some time. For that, food prices increased in the short term. After identifying possible problems, Chinese government immediately sent food security stabilization signals into the market, and promptly introduced a policy of double-season rice cultivation encouragement. With domestic food supply increasing, food prices soon stabilized. In the above case, $p_{2}$ is a function of food supply, food demand, and the produce amount produced by domestic farmers. When import food supply channel closed temporary, $p_{2}$ increase. In our study, the purchase price of food in Changling Village was 4.3 yuan/kg in 2018, and the highest purchase price of food had reached 5 yuan/kg in 2020. From Figure 8, the farmers’ behavioral strategy does not change when there is a little change in the food price.

Then we analyze government’s increase of subsidies for double-season rice cultivation. At this point, m makes changes. From Figure 9, a small change in m does not significantly affect the farmers’ behavioral choice of farmland abandonment. However, it truly stimulates single season rice cultivation to double-season rice cultivation. In this way, the domestic supply increases and the food price stabilized. Based on the analysis above, if the price of agricultural products increases significantly, even small farmland can make more profit. Does it mean that farmers do not need to abandon their cultivated land? Actually not. How much the food price can be increased? 50%, or even 100% or 200% (corresponding to Figure 8). Actually, the increase in farming income is far less than other industries. Moreover, even if the government does not intervene in prices, it is unlikely that prices will rise so much in the short term, as the principle of supply and demand will play in the agricultural market.

Although the weather in Hengyang area has been favorable during the past 20 years, in view of the need of the study, the climate extremes are assumed and analyzed accordingly here. In an extreme case, when climate change leads to a large produce reduction, depending on supply and demand theory, the food price will rise sharply, even reaching the critical value of farmland abandonment. At that time, under the action of market supply and demand, farmers will choose to abandon off-farm work and back to their farmland. Based on analysis above, the model in this paper is a universal model and can be applied to various situations. In particular, $p_{1},\;p_{2},\;m$ etc. can be regarded as a function of other factors, which extends the applicability of this model to some extent.

## 4. Discussion

Based on the analysis of the above evolutionary game model, we learned that farmland abandonment in China is the rational choice of rural households under the background of China’s urbanization and industrialization acceleration if the local government chooses farmland abandonment supervision with the consideration of food security. On the one hand, the cost of supervision is relatively high; on the other hand, rural households have a low cost of dealing with supervision. Under the joint effect of the above two factors, government supervision will easily fall into the “regulatory ineffectiveness trap”.

Compared with the existing studies, the deficiencies of our study are as follows. Firstly, the model of our study is a general model that extracting the factors of farmland abandonment from the existing empirical papers. It is difficult to take into account the heterogeneity and diversity of each region in China. Compared with the existing studies, the highlight of this paper is the analysis of the overall logic, especially the evolution of farmers’ and government’s choices in different situations. Secondly, in order to simplify the study, the model setting of this paper refers to existing studies ([33,37]) and simplifies the abstraction of the real situation. It may deviate from the reality to a certain extent, but it does not affect the overall analytical framework of our study. Thirdly, the equilibrium results of our study only prove that, the existence of heterogeneity in the optimal decisions of farmers and government in different situations. And the equilibrium solution conditions are not completely accurate, it can only provide some rough references, analytical ideas, policy recommendations on farmland abandonment problem for government.

The global research into farmland abandonment mainly stems from concerns about food security. The following is an analysis of the food security problem in China. Under the premise of if we “do not control the population” and “do not protect the existing farmland”, China’s food security problem is a long-term, macroscopic, strategic issue, but not a short-term (e.g., annual, quarterly), micro-decision-making issue. China is an open country. Chinese food supply and demand are regulated by both domestic and international markets. Although for individual small farmers, the farmland in China is limited. But we can’t ignore the huge number of those farming small farmers. At the same time, some large farmers who are suppliers of bulk agricultural products. So that, generally, the total supply is relatively sufficient. In addition, the international market can be used to increase the supply. However, the import of agricultural products is only suitable for a few cases: Firstly, the international price is lower than the domestic price, especially bulk product. In this case China is importer; secondly, a domestic disaster resulting in huge production cuts, which may lead to import; thirdly, China’s resource endowment led to the structural shortage of species, which means the need of import. Thus, the supply of international and domestic markets determines that, the short-term prices of agricultural products (not the long-term trend of ten years, and this trend is not meaningful to farmers’ decisions) cannot rise significantly. And even if there is a volatile rise, it is not enough to change the decisions of farmland abandonment.

In a deeper analysis, since the international price of agricultural products is lower than the domestic price, and it is possible to feed the Chinese people by importing, the question is why do governments still focus on food security, and even supervise farmland abandonment behavior. Actually, a country with a small population may be able to feed people using imports but a big country cannot do this. With 1.4 billion people (which will increase in the long run), if a country relies on imports for more than 20% of its food needs, (not to mention 100%), it will no longer have control of its lifeline. The reason that China can take advantage of international markets, and especially imports of low-cost agricultural products, is that it has sufficient self-supply capacity. It has the farmland, the technology and the farmers in place. Without these, China will not be able to use the international market, but the big suppliers in the international market will have the chance to control China. They can raise prices, stopping the food trade with China, or use this as a bargaining chip to influence the decisions of parties, which has possibly affecting world stability. By the principle of supply and demand in economics, in view of Chinese huge population, there are too many “rice bowls” to feed, so the “rice bowls” must be in Chinese hands. So, to some extent, China will “starve the whole world” if it really lacks a lot of food (mainly the ability to supply)

Based on the above analysis, the solution for food security depends on a reasonable population and high-yielding farmland. Therefore, rather than the useless supervision of farmland abandonment, it is better to control the population, improve the quality of existing farmland and increase production through science and technology. In our research, under the political pressure from central government, local governments have to conduct unreasonable policy. This is clearly contrary to social welfare maximization. Thus, we must determine how to help to ban or reduce this kind of top–down irrational behavior. In addition to changing the one-way information transmission with the construction of a system that aids the formation of a good two-way interaction mechanism between the upper and lower levels of government, it is necessary to take strength from society, such as the public and sociologists, into consideration. Besides, smooth channels of responding to public opinion are also very important, which will help government to receive more useful information regarding relevant policies and make policies more reasonable. In this way, we can rationally allocate resources and improve the overall welfare of society.

Additionally, as mentioned in our introduction, regarding farmland abandonment in China, it is necessary to discuss another kind of farmland abandonment which is less common; that is, farmland abandonment from some “big farmers”. The reasons for this kind of farmland abandonment are more complex. In fact, rural households with a large amount of farmland always choose to scale up their farmland because of their high total revenue, considerable profit margins and substantial government subsidies. Farmland abandonment in large amounts from certain owners mainly belongs to the following situations: firstly, this kind of abandonment may come from a variety of areas or zones that are occupied by governments at all levels, in which part of the farmland is occupied to build agricultural or industrial parties, but the plan may have failed or sufficient industries to develop the plan may be lacking, and as a result, the land had to be abandoned. Secondly, some large state-owned enterprises and government departments rent farmland from farmers or villages collectively to engage in “special agricultural development”.

However, with the implementation of the eight rules of the CPC Central Committee, most of these activities are banned and the corresponding farmland is leased back, which also causes a certain amount of abandonment. Thirdly, some entrepreneurs obtain farmland in the name of capital entry rural areas to defraud various agricultural subsidies. However, the farmland is not reasonably operated, and as a result, farmland abandonment occurs. The exact definition of such “large farmers” should be “large farmland wasters”. It is clear that farmland abandonment from rural households is the result of the normal function of market mechanisms and does not worsen the allocation of resources. However, the behaviors of farmland abandonment from “large farmland wasters” exposes institutional and policy shortcomings, which worsens the allocation of resources and leads to the wastage of farmland resources. Therefore, avoiding farmland abandonment and destruction by “large farmland wasters” through the joint effort of the market and government will be an important research topic in the future.

## 5. Conclusions and Suggestions

Farmland abandonment in China does exist to a certain extent [38,39,40]. Under the consideration of food security, farmland abandonment supervision has been proposed. Under the pressure of the Chinese central government, local governments have introduced a series of policies to reduce farmland abandonment, such as withdrawing farmland after a certain number of years of farmland abandonment and imposing fines for short-term farmland abandonment. However, supervision and fines to alleviate farmland abandonment have produced very few effects in some pilot regions. In the above research, our study examines the evolutionary equilibrium paths and influencing factors of behavioral choices between rural households and the government.

The results of the study show that an increase in farmland income will reduce the tendency of households to abandon their farmland, while an increase in non-farm income will increase the tendency of households to abandon their farmland; however, additional incentives for cultivation will not reverse their behavior. Thus, the key to reducing the current level of farmland wastage and improving food security in China is to increase households’ farmland income. From relevant research, the profitability of food cultivation is encouraging. However, as the amount of farmland belonging to one single household is extremely small in most of the countryside areas in China, the overall total income of one household is extremely small, which leads to household farmland abandonment [41]. Thus, increasing the amount of farmland area owned by a single household and increasing the scale of cultivation is an effective way to increase the total income of food households. Therefore, through land transition, right of ownership changes and other approaches, we can encourage rural households to centralize farmland cultivation instead of engaging in scattered management [42]. Besides, from the perspective of increasing rural households’ income, it is necessary to cultivate the outsourcing service market in order to effectively induce households’ participation in the division of labor [43]. In this way, we can improve the output efficiency of farmland. In the process of large-scale farmland operation, surplus labor should be allowed to flow to the industries with a high input–output ratio in accordance with the supply and demand law of the labor market.

Numerical analysis shows that local governments’ supervision costs and opportunity costs have become a relevant and important reason for the government to give up supervision, but the pressure from the central government pushes local government to insist upon supervision. Under the consideration of food security, the government has adopted strict supervision measures for farmland abandonment. However, due to the inherent mechanism of farmland abandonment and the characteristics of rural households’ behavior, supervision is difficult and has high costs, which makes supervision basically ineffective and causes governments to even fall into the “regulatory trap”. The results of statistical analysis also confirm our conclusions. China does have long-term, strategic and macro food security problems. These are caused by the contradiction between population quantity and farmland resources and are unrelated to farmland abandonment or farmland abandonment supervision. It should be regarded as a blessing if farmland abandonment occurs due to the domestic and foreign market mechanisms, and the choice of farmers is based on comparative interests without affecting China’s food supply. When we can regard “abandonment” as the land becoming “fallow” and thus improving the quality of farmland, governments’ farmland abandonment supervision then becomes completely unnecessary [44,45,46,47].

Therefore, according the research and relevant studies [31,48], we make the following recommendations. Firstly, governments need to change the concept of food security; farmland abandonment does not mean farmland loss. China’s food security is general and long-term; there is a contradiction between population quantity and farmland but not in the sense of short-term farmland abandonment. Farmland abandonment not only does not affect food security, but rather indicates short-term food security. Secondly, the government should abandon the supervision and punishment policy of farmland abandonment determined by the market mechanism, because it is neither necessary nor extremely efficient, and sometimes even causes governments to fall into a regulatory trap. Last but not least, it is necessary to optimize farmland protection policies. Farmland can be abandoned, but it cannot be destroyed or used for other purposes; the utilization rate of construction land should be improved and as little farmland should be occupied as possible. Restoring the balance of construction land and the consolidation of farmland, while strictly grasping the implementation of quantity and quality, is important. Finally, the government should improve the quality of farmland and expand its area. The improvement of inferior land is very important to China’s farmland reserves. Protecting farmland, developing agricultural technology and controlling the population can guarantee food security in a fundamental and long-term way.

## Figures and Tables

**Table 1:** Symbols and descriptions.

**Symbols**	**Meaning and Description**
α	The probability that a household chooses to abandon farmland; the probability that it does not is 1−α,0≤α≤1
β	The probability that a government chooses to supervise the use of farmland; the probability that it does not is 1−β,0≤β≤1
$r_{1}$	Proceeds from farmland abandonment by landowning households given to other businesses, such as income from working in urban areas
$r_{2}$	Expected benefits of local government supervision with incentives from government
$r_{3}$	Income from the government stopping the supervision of farmland abandonment and investing the associated funds in other performance-related projects
$r_{4}$	Fines gained from government-supervised land abandonment are equal to the loss of fines for households under the same conditions
$c_{0}$	The cost of local government supervision of abandoned farmland
$c_{1}$	The cost of local governments’ supervision, fines and confiscation for farmland abandonment (e.g., maintenance costs, supervised costs, etc.)
$c_{2}$	The penalty cost of local governments being penalized by the superior governments for the negligent supervision of farmland abandonment
$p_{1}$	Households’ cost of buying foods after farmland abandonment
S	Farmland subsidies received by households
η	The probability that a household will be discovered when abandoning their land
$p_{2}$	Households’ net income from food production
m	The additional bonus that households may receive for growing food under the governments’ supervision

**Table 2:** Behavioral choices of households and the government in the supervised game of farmland abandonment and the profitability matrix.

	Government	Supervision	No Supervision
Household			
Abandonment		$\left(r_{1}-p_{1}+s-\eta r_{4},\eta r_{4}+r_{2}-c_{1}-c_{0}\right)$	$\left(r_{1}-p_{1}+s,r_{3}-c_{2}\right)$
No abandonment		$\left(p_{2}+s+m,r_{2}-c_{0}\right)$	$\left(p_{2}+s,r_{3}\right)$

**Table 3:** Specific values of $a_{11},a_{12},a_{21},a_{22}$ at the local balance point.

Equilibrium Points	$a_{11}$	$a_{12}$	$a_{21}$	$a_{22}$
$E_{1}=(0,0)$	$-p_{1}-p_{2}+r_{1}$	0	0	$-c_{0}+r_{2}-r_{3}$
$E_{2}=(0,1)$	$-m-p_{1}-p_{2}+r_{1}-\eta r_{4}$	0	0	$c_{0}-r_{2}+r_{3}$
$E_{3}=(1,0)$	$p_{1}+p_{2}-r_{1}$	0	0	$-c_{0}-c_{1}+c_{2}+r_{2}-r_{3}+\eta r_{4}$
$E_{4}=(1,1)$	$m+p_{1}+p_{2}-r_{1}+\eta \cdot r_{4}$	0	0	$c_{0}+c_{1}-c_{2}-r_{2}+r_{3}-\eta r_{4}$
$E_{5}=(\alpha^{*},\beta^{*})$	A	0	0	B

## Data Availability

The data in this article comes from the official website of the National Bureau of Statistics of China, http://www.stats.gov.cn/.

## References

[1] He G., Zhao Y., Wang L., Jiang S., Zhu Y. (2019). China’s food security challenge: Effects of food habit changes on requirements for arable land and water. J. Clean. Prod..

[2] Luo B. (2018). 40-year reform of farmland institution in China: Target, effort and the future. China Agric. Econ. Rev..

[3] Singh S., Kumar R., Panchal R., Tiwari M.K. (2020). Impact of COVID-19 on logistics systems and disruptions in food supply chain. Int. J. Prod. Res..

[4] Pu M., Zhong Y. (2020). Rising concerns over agricultural production as COVID-19 spreads: Lessons from China. Glob. Food Secur..

[5] Smith C.J. (2019). China’s Population: Resistance, Compliance, and the National Interest.

[6] Yao Z., Zhang L., Tang S., Li X., Hao T. (2017). The basic characteristics and spatial patterns of global cultivated land change since the 1980s. J. Geogr. Sci..

[7] Chen Y.F., Wang Y.K., Fu B., Wang H.W., Wang W. (2018). Spatial patterns of farmland abandonment and its impact factors in the central Three Gorges Reservoir Area. J. Mt. Sci.

[8] Li S., Li X. (2017). Global understanding of farmland abandonment: A review and prospects. J. Geogr. Sci..

[9] Xu D., Deng X., Guo S., Liu S. (2019). Labor migration and farmland abandonment in rural China: Empirical results and policy implications. J. Environ. Manag..

[10] Sun L., Yan J., Yang Z., Li Z., Xin L. (2016). Drivers of cropland abandonment in mountainous areas: A household decision model on farming scale in southwest china. Land Use Policy.

[11] He Y., Xie H., Peng C. (2020). Analyzing the behavioural mechanism of farmland abandonment in the hilly mountainous areas in China from the perspective of farming household diversity. Land Use Policy.

[12] Zhu X., Xiao G., Zhang D., Guo L. (2020). Mapping abandoned farmland in China using time series MODIS NDVI. Sci. Total Environ..

[13] Zhou H., Yan J., Lei K., Wu Y., Sun L. (2020). Labor migration and the decoupling of the crop-livestock system in a rural mountainous area: Evidence from Chongqing, China. Land Use Policy.

[14] Xie H., Wang P., Yao G. (2014). Exploring the dynamic mechanisms of farmland abandonment based on a spatially explicit economic model for environmental sustainability: A case study in Jiangxi Province, China. Sustainability.

[15] Wang H., Zhang G.H., Li N.N., Zhang B.J., Yang H.Y. (2018). Soil erodibility influenced by natural restoration time of abandoned farmland on the Loess Plateau of China. Geoderma.

[16] Shi T., Li X., Xin L., Xu X. (2018). The spatial distribution of farmland abandonment and its influential factors at the township level: A case study in the mountainous area of China. Land Use Policy.

[17] Deng X., Zeng M., Xu D., Qi Y. (2020). Does social capital help to reduce farmland abandonment? Evidence from big survey data in rural China. Land.

[18] Du J., Zeng M., Xie Z., Wang S. (2019). Power of agricultural credit in farmland abandonment: Evidence from rural China. Land.

[19] Wang Y., Li X., Xin L., Tan M. (2020). Farmland marginalization and its drivers in mountainous areas of China. Sci. Total Environ..

[20] Yang H. (2020). Does rural labor migration have an impact on the area of farmland abandonment in China?. Ph.D. Thesis.

[21] Zhang X., Zhao C., Dong J., Ge Q. (2019). Spatio-temporal pattern of cropland abandonment in China from 1992 to 2017: A meta-analysis. Acta Geogr. Sin..

[22] Zhang Y., Li X., Song W., Zhai L. (2016). Land abandonment under rural restructuring in China explained from a cost-benefit perspective. J. Rural Stud..

[23] Liang X., Li Y., Zhou Y. (2020). Study on the abandonment of sloping farmland in Fengjie County, Three Gorges Reservoir Area, a mountainous area in China. Land Use Policy.

[24] Yu Z., Liu L., Zhang H., Liang J. (2017). Exploring the factors driving seasonal farmland abandonment: A case study at the regional level in Hunan Province, central China. Sustainability.

[25] Wang E.T., Hu H.F., Hu P.J.H. (2013). Examining the role of information technology in cultivating firms’ dynamic marketing capabilities. Inf. Manag..

[26] Hou J., Fu B., Liu Y., Lu N., Gao G., Zhou J. (2014). Ecological and hydrological response of farmlands abandoned for different lengths of time: Evidence from the Loess Hill Slope of China. Glob. Planet. Chang..

[27] Huang Y., Li F., Xie H. (2020). A scientometrics review on farmland abandonment research. Land.

[28] Norse D., Ju X. (2015). Environmental costs of China’s food security. Agric. Ecosyst. Environ..

[29] Kang Y. (2019). Food safety governance in China: Change and continuity. Food Control.

[30] Guo Z., Bai L., Gong S. (2019). Government regulations and voluntary certifications in food safety in China: A review. Trends Food Sci. Technol..

[31] Lichtenberg E., Ding C. (2008). Assessing farmland protection policy in China. Land Use Policy.

[32] Gao S., Ling S., Liu X., Dou X., Wu R. (2020). Understanding local government’s information disclosure in China’s environmental project construction from the dual-pressure perspective. J. Clean. Prod..

[33] Gao S., Ling S., Liu W. (2018). The role of social media in promoting information disclosure on environmental incidents: An evolutionary game theory perspective. Sustainability.

[34] Friedman D. (1998). On economic applications of evolutionary game theory. J. Evol. Econ..

[35] Friedman D. (1991). Evolutionary games in economics. Econometrica.

[36] Ginits H. (2009). Game Theory Evolving.

[37] Feng J., Yao J., Zhang K. (2018). Operation mechanism of PPP project of farmland water conservancy: Based on stochastic evolution game. J. Technol. Econ..

[38] Lu C. (2020). Does household laborer migration promote farmland abandonment in China?. Growth Chang..

[39] Xiao G., Zhu X., Hou C., Xia X. (2019). Extraction and analysis of abandoned farmland: A case study of Qingyun and Wudi counties in Shandong Province. J. Geogr. Sci..

[40] Wei S., Ying Z. (2019). Farmland abandonment research progress: Influencing factors and simulation model. J. Resour. Ecol..

[41] Huang S., Guo D., Wu J. (2019). An evaluation of the effects of direct food subsidy policy. Chin. Rural Econ..

[42] Ma L., Long H., Tu S., Zhang Y., Zheng Y. (2020). Farmland transition in China and its policy implications. Land Use Policy.

[43] Wang B., Zhang G.H., Shi Y.Y., Zhang X.C., Ren Z.P., Zhu L.J. (2013). Effect of natural restoration time of abandoned farmland on soil detachment by overland flow in the Loess Plateau of China. Earth Surf. Process. Landf..

[44] Peng W.Y., Zhang K.L., Chen Y., Yang Q.K. (2005). Research on soil quality change after returning farmland to forest on the loess sloping croplands. J. Nat. Resour..

[45] Li J., Liu Y., Hai X., Shangguan Z., Deng L. (2019). Dynamics of soil microbial C:N:P stoichiometry and its driving mechanisms following natural vegetation restoration after farmland abandonment. Sci. Total Environ..

[46] Zhang C., Liu G., Xue S., Wang G. (2016). Soil bacterial community dynamics reflect changes in plant community and soil properties during the secondary succession of abandoned farmland in the Loess Plateau. Soil Biol. Biochem..

[47] Zhong Z., Zhang X., Wang X., Fu S., Wu S., Lu X., Yang G. (2020). Soil bacteria and fungi respond differently to plant diversity and plant family composition during the secondary succession of abandoned farmland on the Loess Plateau, China. Plant Soil.

[48] Chen W., Ye X., Li J., Fan X., Liu Q., Dong W. (2019). Analyzing requisition–Compensation balance of farmland policy in China through telecoupling: A case study in the middle reaches of Yangtze River Urban Agglomerations. Land Use Policy.

